# The effect of sleep deprivation on postural stability among physically active young adults

**DOI:** 10.1038/s41598-023-44790-4

**Published:** 2023-10-14

**Authors:** Rafał Stemplewski, Julia Ciążyńska, Magdalena Cyma-Wejchenig, Janusz Maciaszek

**Affiliations:** 1 Department of Digital Technologies in Physical Activity, Poznań University of Physical Education, Poznań, Poland; 2 Department of Physical Activity and Health Promotion Science, Poznań University of Physical Education, Poznań, Poland

**Keywords:** Health care, Risk factors, Musculoskeletal system

## Abstract

The study aimed to evaluate the effect of sleep deprivation on postural stability among physically active young adults. The study involved 22 physical education students. Average velocities and spatial distribution of the center of pressure displacements were taken as indicators of postural stability (double and one-leg standing). Two-way ANOVA with two factors of repeated measurements—“session” (control-experimental) and “daytime” (evening-morning)—was used. For indicators of the spatial distribution of the center of pressure in double stance with eyes open and eyes closed, and for average velocities for measurements with eyes closed, statistically significant interaction effects were found (at least *p* < 0.01, ƞ^2^ > 0.36, power statistics > 0.90) with the general tendency of higher results in the morning in the session with sleep deprivation than in the control session. In one-leg standing, an increase of average velocities was observed in the control session, and no differences in the session with sleep deprivation (interaction effect: at least *p* < 0.01, ƞ^2^ > 0.37, power statistics > 0.90). Besides spatial distribution indicators in double stance, there were no statistical differences between evening-morning tests in the session with sleep deprivation. Despite significant interaction effects, only the results of spatial distribution indicators in double stance were higher in the morning than in the evening in the session with sleep deprivation. So, no clear decline in postural stability after sleep deprivation was observed. This may suggest that sleep deprivation prevents natural regeneration rather than significantly worsening postural stability among physically active adults. It’s possible that systematic physical activity might be one of the factors decreasing the risk of accidents among people exposed to sleep deprivation.

## Introduction

Sleep deprivation (SDep) is indicated as one of the leading causes of fatalities and injuries at work and in transportation^1,2^ which might be connected to the worsening of postural stability (PS) in prolonged wakening^3–5^. Maintaining upright body posture during standing as well as during other daily activities depends on the integration of visual, proprioceptive, and vestibular inputs^6^. The phenomenon is associated with different terminology—mainly, body balance and PS. Both are classically defined in the context of the possibility of maintaining the vertical projection of the center of gravity (COG)—or gravity line—inside the base of support, or as angle deviation from the vertical line with the rotation axis in the ankle joint^7–9^. The interpretation is connected to the inverted pendulum model proposed by Winter et al.^10^ assuming the human body as a rigid object pivoting around the ankle^11^. The position of the human body is never still and COG, analyzed in a two-dimensional Cartesian system, oscillates around the optimal position which is called postural sway^6,7^. Wider and faster postural sway is usually interpreted as worse body balance^12^. Nowadays, the model is considered to have limitations in assuming the human body acts as a rigid object above the ankle joint to the head. More recent studies have proposed the double-inverted pendulum model^13^. However, adapting it for analysis to estimate the relative contribution of each joint is still challenging. Although the single inverted pendulum model excludes hip motion and ankle-hip interaction, it is functionally correct and practically acceptable for experimental studies using posturography for postural sway analysis^14^. It should be mentioned that PS might be also defined as the ability to counteract the destabilization factors of internal or external origins. However, both terms are very often used interchangeably in studies exploring the same phenomenon with the same biomechanical indicators.

Considering that COG monitoring is challenging, simplified assessment is used in most studies, based on posturographic analysis of the center of pressure (COP) displacements reflecting muscle forces generated to maintain COG close to the optimal position. COP oscillates around the vertical projection of the COG position and the difference between them is proportional to the acceleration of COG. It means that more rapid shifts of COG make the central nervous system (CNS) generate adequately higher forces to counteract^10^. Expanding the theory, the COP-COG difference might reflect the postural control process—the lower the difference, the better the control^15^. Maintaining COG projection inside the base of support is possible with the integration of mentioned sensory inputs and producing appropriate reactions of postural muscles which is the role of CNS and is connected to a process called postural control^16^. It was formerly indicated that during undisturbed standing the regulation of body posture is dependent mostly on fast, monosynaptic reflexes at the spinal cord level, released in feedback mode^10,17^. However, more recent studies have shown that posture control tends to be based on feedforward processing—the integration of sensory inputs and the use of internal models in the brain to produce reflexes of higher latency. The process might be located in the cerebellum and be similar to nervous control of movement involving anticipatory postural adjustments^18,19^. Furthermore, during more demanding activities or external situations—a higher level of the CNS—including mainly the cerebellum, basal ganglia, thalamus, and cerebral cortex^20–22^—is activated to produce reactions of higher latency. So, proper functioning of the CNS is crucial in maintaining upright body posture. When the CNS is affected by external demands—such as anxiety, e.g. at-hight work^23,24^ or physical^6^ and mental fatigue^25^—as well as internal demands like degeneration connected to involution processes^26^ or cognitive demand such as during dual-task^27^ this might have a significant impact on PS, and, in consequence, on falls/accidents.

One of the factors that might affect PS is SDep. Some studies have analyzed this phenomenon and higher values of average velocities, ranges, surface area, and other indicators of COP displacements in SDep conditions were observed^12,28,29^ in posturographic analyses. The effects were reported to be stronger in stance with eyes closed^3,12^. Some researchers indicated that the observed changes differed according to time-of-day^4^. The mechanisms of the phenomenon are still unclear^30^. It has been posited that a potential role could be played by reduced adaptation abilities and lapses in attention^31^ or modulation by attentional resources^12^. It has also been demonstrated that muscle strength, which is important for PS, is decreased after a prolonged time of waking^32,33^, and in turn, this might be associated with decreased body temperature^34^. In a recent review, Pillard^2^ mentioned that the possible influence of SDep on PS might be even wider through functions connected to: (i) information taking system (vision, ocular motor function, vestibulo-ocular system, and sensory reweighting), (ii) decision-making system (brain and executive functions), (iii) motor execution system (spinal and neuromuscular functions). However, the author concluded the that negative effect of SDep on these functions requires further exploration. It is well known that systematic physical activity (PA) increases physical fitness including PS^35,36^ and increases the ability to counteract fatigue^37,38^. In one previous study, it was found that a decrease in PS after physical effort was lower among physically active men than among inactive ones^39^. To the best of the authors’ knowledge, no previous studies have assessed the effect of SDep on PS among physically active people. If SDep is connected to fatigue^3^ it would be interesting to see if physically active young adults are resistant to a decrease in PS after prolonged awakening. So, this study aimed to check the magnitude of SDep effects on PS among physically active young adults.. In the context of previous research, it is difficult to offer an univocal hypothesis connected to the effect of SDep on PS among young, physically active people. However, taking into account the above-mentioned positive influence of PA on PS and the ability to counteract fatigue, the authors lean towards the assumption that SDep has a minor or non-existent effect on PS among adults who are physically active.

Moreover, the secondary aim was to check the difference in PS change that occurs during the night—the evening-morning difference after sleep vs. the evening-morning difference after SDep. Most of the previous studies in this area took experimental models connected to analysis differences between measurements of PS after night with or without sleep (and at some time points of the following day)^4,12,29,31,40^. It was hypothesized that posturographic parameters are lower (better PS) after a night of sleep and remain unchanged after a night without sleep in comparison to evening values. To the best of the authors’ knowledge, this is the first study that compares evening-morning changes after SDep as well as analyzes the differences in morning-evening changes between sleep and SDep states. The obtained results might extend existing knowledge connected to the SDep effect on PS.

## Methods

### Study design

**Figure 1:**
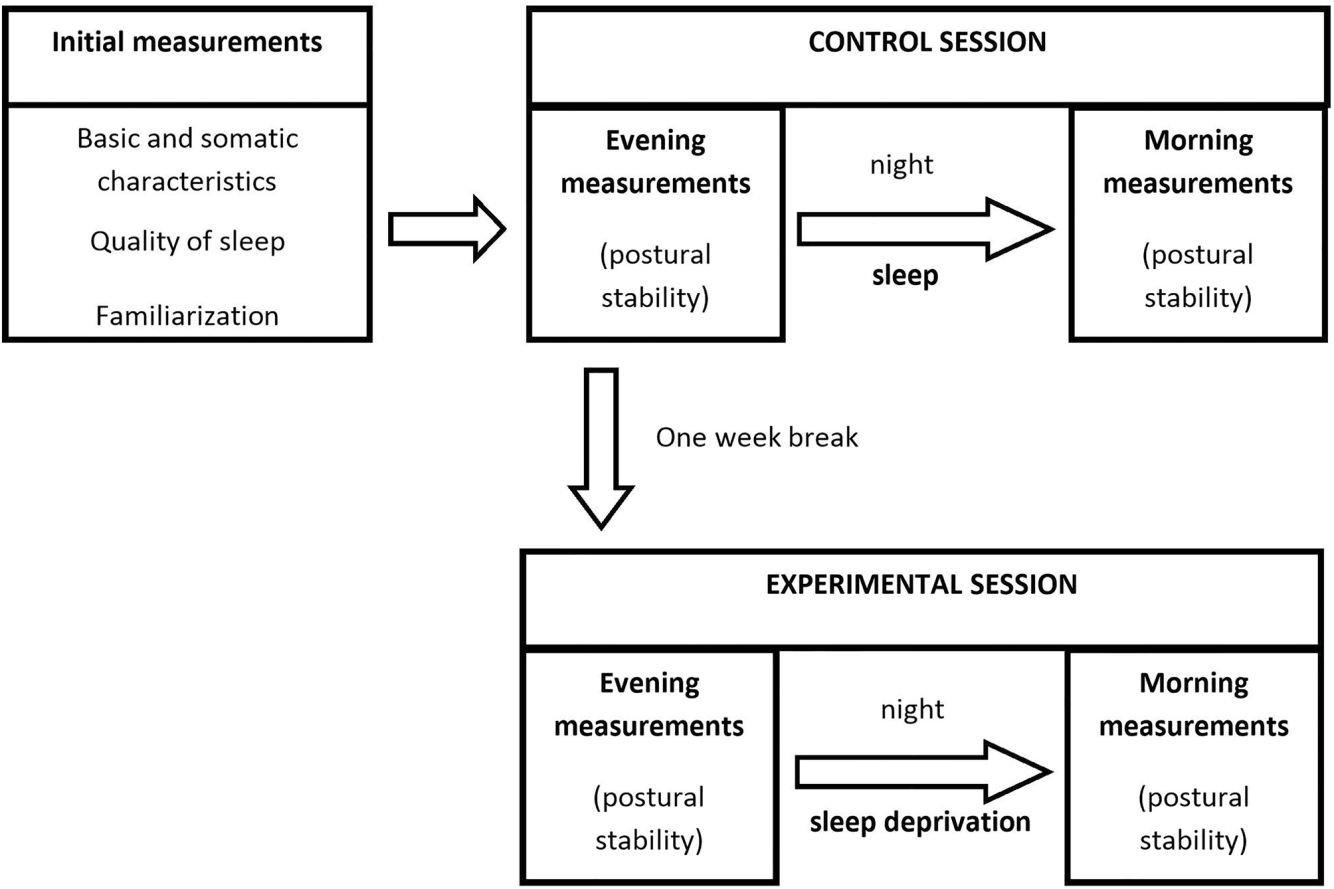
Schema of study design.

The experiment was designed as a one-center within-group study^4,12^ conducted at the Poznań University of Physical Education in Poland. All measurements were made in the Laboratory of Human Motoric with access to appropriate equipment. The main measurements of dependent variables were carried out in two separate sessions: control (without sleep deprivation) and experimental (with sleep deprivation) with a week break between them to avoid the learning process. In each session, two measurements were done—in the evening (6.00–8.00 pm) and the morning (6.00–8.00 am) of the next day. Before the experiment, initial measurements connected to basic and somatic characteristics assessments, as well as information about sleep quality were taken. Participants were also familiarized with measurement methods (Fig. 1).

### Participants

The study began with the 27 first-year students of physical education qualified according to eligibility criteria. The study protocol was based on voluntary participation in four measurements (two measurements per session). Five of the subjects failed to attend one of the measurements. Finally, the research was carried out on a group of 22 participants (16 men and 6 women). All subjects were healthy and presented a high level of fitness and PA. Every candidate for physical education must undergo a medical examination to exclude potential counterindications to undertaking PA of moderate and high intensity. As a weekly routine, students of the first year have on average 7 h of different sports activities such as basketball, volleyball, soccer, handball, field hockey, track and field, gymnastics, and swimming. Taking into account only this University routine, they complete over 400 min per week of moderate-to-vigorous PA. In the context of WHO recommendations for adults^41^, this level of PA may bring additional health benefits. None of the participants declared serious sleep disorders.

Inclusion criteria were based on the subjects’ declaration. Candidates with regular sleeping habits, lack of nervous system disorders, bone fractures, or muscle injuries 12 months before the examination, gambling habits, and use of medication that might influence PS (e.g. diuretics, psychotropics, or sedatives) were included in the study. There were no age limitations (however, participants were first-year students, so age was around 20 years), gender, and skill level. Exclusion criteria were serious sleep disorders, acute illness, as well as any diseases, injuries, or medication use that might influence PS.

Subjects were informed in detail about the study procedures and gave their written informed consent for the experimental procedure. Participation in the experiment was voluntary. The study was approved by the Bioethics Committee of the Poznan University of Medical Sciences (decision no. 989/17) and was in line with the Helsinki Declaration^42^.

### Intervention

During the experimental session, SDep was introduced as the intervention. On an experimental day, participants were asked to wake up between 6.00 am and 7.00 am, and make their ordinary routine as classes, work, etc., for the whole day^5^ avoiding the use of alcohol, coffee, cigarettes, or any stimulants^4^. In the evening (6.00–8.00 pm) they came back to the University and underwent measurements.

Then subjects were asked to stay in the hall of the main building of the University for the whole night. They were allowed to play cards, board games, console games, read, chat, etc.^4,5^. None of them could leave the building. Every full hour, one of the researchers checked whether every participant was present and awake. At 6.00 am the morning measurements started. Each participant stayed awake for 24 h.

### Primary outcomes

Postural stability as a dependent variable was examined with the use of the posturography method based on the measurement of COP displacements.

#### Instrumentation and signal processing

The AccuGait-Optimized force platform (AMTI ACG-O model, AMTI Watertown, MA) was used for collecting COP data during tests. The platform was equipped with strain gauges that facilitated the monitoring of the changes in ground reaction forces. It was connected to a computer equipped with Balance Clinic software provided by the manufacturer. Based on ground reaction forces data, the position and displacements of COP were estimated by the software.

A sampling frequency of 100 Hz was used during data acquisition. Raw data were low-pass filtered (the fourth-order Chebyshev II filter with a cut-off frequency of 10 Hz) to remove noise from the obtained signal of COP displacements^43^.

#### Trials and outcomes

The force platform was placed on a hard and flat floor surface. Before the start of the testing procedures, the participants rested in a sitting position for 5 min. During the measurement, only the researcher and the participant were present in the room. Participants were directed to perform tests in three versions:

1. Double stance with eyes open (EO);
2. Double stance with eyes closed (EC);
3. One-leg standing with eyes open (OLS).

Each test was run two times to give a total of 6 trials in each measurement with a 20-s break between the following trials. The order of trials was random to avoid potential learning effects. Randomization was performed before each measurement with the use of original software written in Python. An average of two repetitions of specific tests was taken as the final result.

The subjects were asked to stand barefoot on the force platform and remain still with the upper limbs positioned at the side of the body. For double stance, the feet were placed in a position similar to their natural stance—about 30 degrees to each other and approximately 5 cm between the heels^44^. In the case of OLS participants placed the dominant foot in the center of the platform.

The primary outcomes of the study were:

a) Average velocity of COP displacements and its components in anterior–posterior (AP) and medio-lateral (ML) directions (Vavg, VavgAP, VavgML, respectively). This was calculated as a ratio of the total path length covered by COP during the test, and the time of the test (cm/s).

b) Indicators of the spatial distribution of COP displacements—ranges in AP and ML directions and ellipse area of COP displacements (RangeAP, RangeML, Area95%, respectively). Ranges were calculated as differences between maximal forward–backward and leftward-rightward positions of COP (cm) along the Y and X axis in the Cartesian coordinate system, respectively. The ellipse area covered 95% of each recorded point of COP displacements during the test (cm^2^).

Average velocities, ranges, and sway area of COP displacements are commonly used posturographic indicators of postural stability^45^. The average results of the two trials of the posturographic test are sufficient to obtain values of the Intraclass Correlation Coefficient above 0.9, at least in the case of the average velocity of COP displacements^46^. As the standard interpretation, it was assumed the increased results of velocity and spatial distribution of COP displacement values as the indicators of decline in PS level^12,39,40^.

### Secondary outcomes

The study population was characterized by age, body weight and height, and BMI (calculated as body weight/height^2^ [kg/m^2^]). The subjects were also asked to subjectively assess the quality of their sleep within a few days before the examination with possible answers of “very poor”, “poor”, “average”, “good”, and “very good”. Moreover, daytime sleepiness was screened using an Epworth Sleepiness Scale (ESS)^47^—modified in 1997. This is composed of 8 questions with a possible 4-point rating (0–3) scale for answers. The result of the ESS is a sum of ratings from each answer. The standard interpretation of results is as follows: 0–5 Lower Normal Daytime Sleepiness, 6–10 Higher Normal Daytime Sleepiness, 11–12 Mild Excessive Daytime Sleepiness, 13–15 Moderate Excessive Daytime Sleepiness, and 16–24 Severe Excessive Daytime Sleepiness. The ESS is commonly used and translated for many languages with high indicators of validity and reliability (https://epworthsleepinessscale.com/about-the-ess/). A license was granted to use the ESS form: Mapi Research Trust, Lyon, France, https://eprovide.mapi-trust.org.

### Sample size

Taking into account the one-factor within effect in repeated measures (lack of possibility to calculate interaction effect for study structure with two within factors of repeated measures), the range of the number of subjects was estimated to equal 26 with power statistics = 0.8, alpha level = 0.05, and assuming strong effect size = 0.14 (according to Cohen’s classification). The same value of sample size was estimated for the within-between structure of an experiment. Calculations were made with the use of G*Power v. 3.1.9.6 (Franz Faul, Christian-Albrechts-Universitat, Kiel, Germany) software.

### Statistical analysis

The main calculations related to the assessment of the variability of dependent variables were done based on the two-way analysis of variance (ANOVA). Variables were checked with the use of the Shapiro–Wilk test to estimate the normality of distribution. It was found that in most of the cases, distribution didn’t differ significantly from the normal one. On the other hand, an analysis of variance is quite robust for violation of the condition of normality^48^. Taking into account two repeated measurements the condition of sphericity wasn’t relevant.

The analysis was applied taking into account two within-group factors of repeated measurements, both with two levels: “session” (control and experimental), and “daytime” (evening and morning). For interaction effects (“session” × ”daytime”) the eta-square as effect size was calculated. The effect size indicates the percent of variance explained by particular effects of the dependent variable. To compare the average values of average velocities, ranges, and area of COP displacement (both evening-morning within each session and between sessions for evening and morning) Bonferroni detailed post-hoc comparisons were used. The minimum level of statistical significance was defined as *p* ≤ 0.05. The study was conducted using the Statistica v. 13.0 software program (TIBCO Software Inc., Palo Alto, CA, USA).

## Results

### Results of initial measurements

Basic characteristics for age, somatic parameters, and daytime sleepiness are shown in Table 1.

**Table 1:** Average values, and standard deviations for age, somatic parameters and daytime sleepiness in study group.

	Women (n = 6)	Men (n = 16)	All subjects (n = 22)
	$$\overset{\lower0.5em\hbox{$\smash{\scriptscriptstyle\leftharpoonup}$}}{\text{x}} \pm {\text{SD}}$$	$$\overset{\lower0.5em\hbox{$\smash{\scriptscriptstyle\leftharpoonup}$}}{\text{x}} \pm {\text{SD}}$$	$$\overset{\lower0.5em\hbox{$\smash{\scriptscriptstyle\leftharpoonup}$}}{\text{x}} \pm {\text{SD}}$$
Age (years)	20.2 ± 1.17	20.2 ± 0.66	20.2 ± 0.80
Height (cm)	168.6 ± 2.56	182.8 ± 6.44	179.0 ± 8.56
Weight (kg)	65.8 ± 16.92	83.8 ± 17.99	78.9 ± 19.15
BMI (kg/m^2^)	23.1 ± 5.23	25.0 ± 4.36	24.4 ± 4.56
ESS (points)	8.3 ± 2.34	8.8 ± 2.86	8.7 ± 2.67

ESS, Epworth Sleepiness Scale.

The mean age of the study group was 20.2 ± 0.80 years with very similar values in men and women. BMI for the whole group was equal to 24.4 kg/m^2^ on average. The lowest value was observed among women (19.1 kg/m^2^), and the highest among men (36.1 kg/m^2^). Daytime sleepiness was scored as normal or mild sleepiness among the group. The average value for the whole group was 8.7 ± 2.67 points which is interpreted as higher normal daytime sleepiness. In the case of subjective assessment of sleep quality within a few days before the examination, participants declared very good or good sleep (63.6% and 36.4%, respectively).

### Results of postural stability measurements

**Figure 2:**
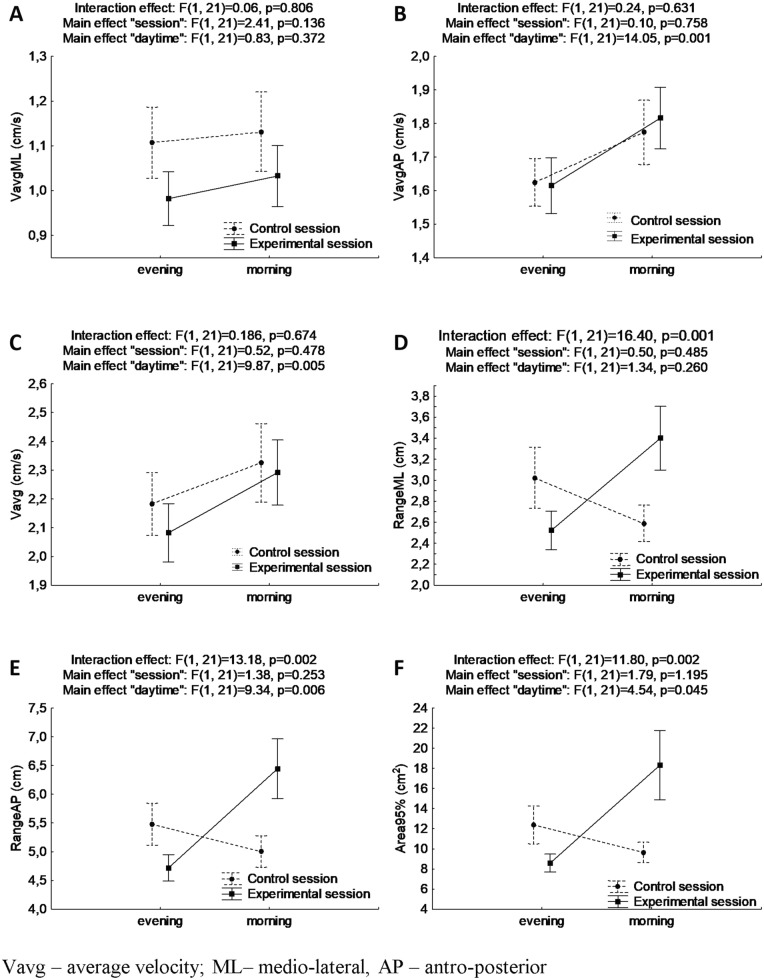
Mean and standard error of measurement values of postural stability indices in double stance with eyes open for evening and morning trials during control and experimental session, and results of two-way ANOVA with repeated measures (**A**, **B**, **C**, **D**, **E**, **F** for VavgML, VavgAP, Vavg, RangeML, RangeAP, Area95%, respectively).

Results for ANOVA with repeated measures for postural stability indicators in trials of EO are presented in Fig. 2.

There were no statistically significant interaction effects “session” × ”daytime” observed for average velocities. In the case of VavgAP and Vavg, it was found significant main effects of “daytime” (F_(1, 21)_ = 14.05, *p* = 0.001, ƞ^2^ = 0.40, power statistics = 0.95, and F_(1, 21)_ = 9.87, *p* = 0.005, ƞ^2^ = 0.32, power statistics = 0.85, respectively).

There were observed statistically significant interaction effects “session” × ”daytime” for RangeML, RangeAP, and Area95% (F_(1, 21)_ = 16.40, *p* = 0.001, ƞ^2^ = 0.44, power statistics = 0.97, and F_(1, 21)_ = 13.18, *p* = 0.002, ƞ^2^ = 0.39, power statistics = 0.93, and F_(1, 21)_ = 11.80, *p* = 0.002, ƞ^2^ = 0.36, power statistics = 0.91, respectively). Values of RangeML, RangeAP, and Area95% were higher in the morning in the experimental session (which might be interpreted as worse PS) than in the control session (*p* = 0.012, *p* = 0.018, and *p* = 0.017, respectively). Comparison between morning and evening measurements in the experimental session revealed an increase of values (which might be interpreted as a decline in PS) after prolonged awakening (Bonferroni post-hoc *p* = 0.006, *p* = 0.004, and *p* = 0.006, respectively).

The main effects of “daytime” were also found for RangeAP and Area95% (F_(1, 21)_ = 9.33, *p* = 0.006, ƞ^2^ = 0.30, power statistics = 0.83, and F_(1, 21)_ = 4.54, *p* = 0.045, ƞ^2^ = 0.18, power statistics = 0.53, respectively).

**Figure 3:**
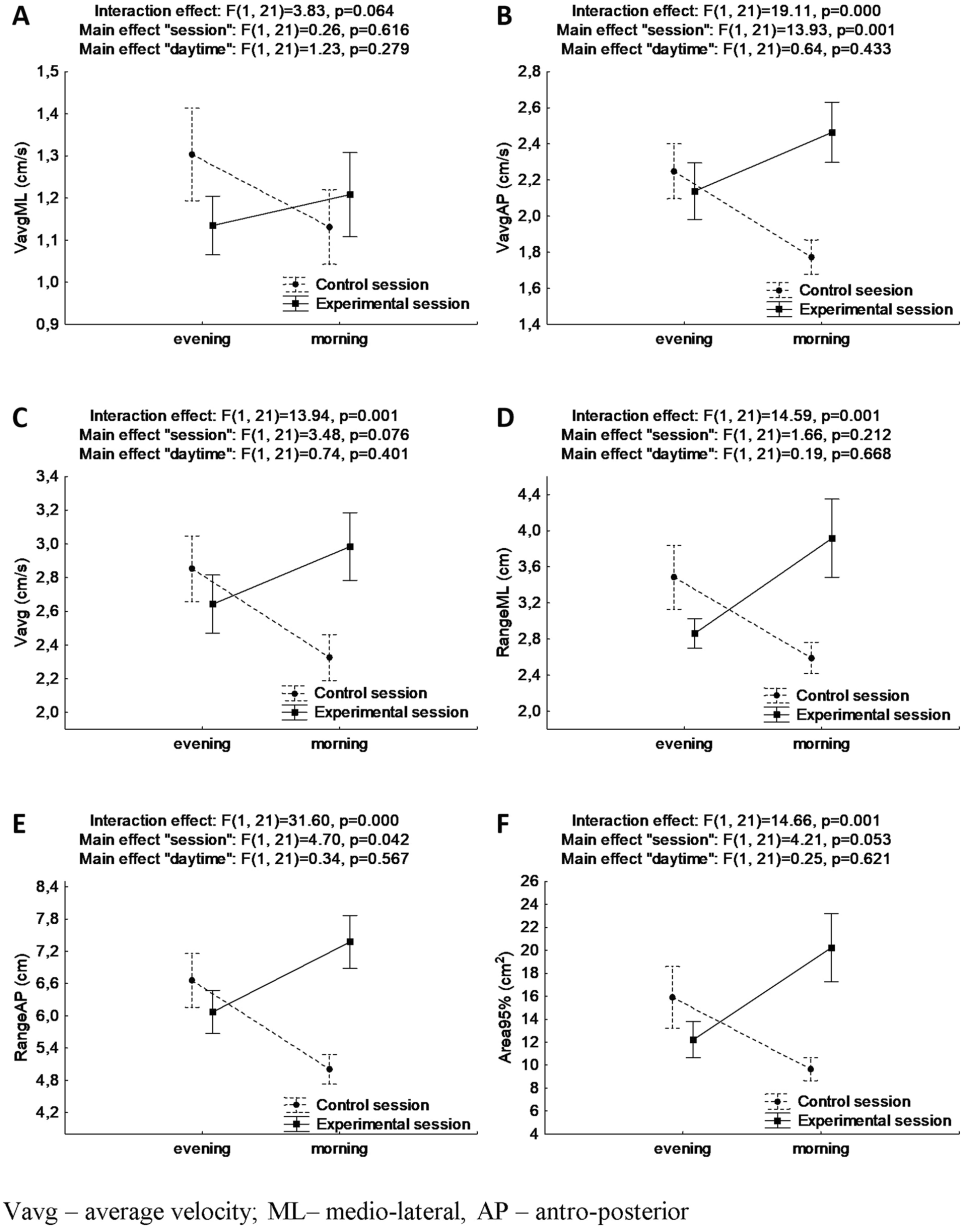
Mean and standard error of measurement values of postural stability indices in double stance with eyes closed for evening and morning trials during control and experimental session, and results of two-way ANOVA with repeated measures (**A**, **B**, **C**, **D**, **E**, **F** for VavgML, VavgAP, Vavg, RangeML, RangeAP, Area95%, respectively).

Results for ANOVA with repeated measures for postural stability indicators in trials of EC are presented in Fig. 3.

In the case of VavgAP and Vavg, it was found significant interaction effects of “session” x “daytime” (F_(1, 21)_ = 19.11, *p* < 0.001, ƞ^2^ = 0.48, power statistics = 0.99, and F_(1, 21)_ = 13.94, *p* = 0.001, ƞ^2^ = 0.40, power statistics = 0.94, respectively). Bonferroni's detailed post-hoc analysis revealed higher values of VavgAP and Vavg (which might be interpreted as worse PS) in the morning in the experimental session than in the control session (*p* < 0.001 and *p* = 0.004, respectively) as well as lower values (which might be interpreted as better PS) in the morning than in the evening for control session (*p* = 0.009 and *p* = 0.026).

It was also observed the main effect of “session” for VavgAP (F_(1, 21)_ = 13.93, *p* = 0.001, ƞ^2^ = 0.40, power statistics = 0.94).

There were observed statistically significant interaction effects “session” × ”daytime” for RangeML, RangeAP and Area95% (F_(1, 21)_ = 14.59, *p* = 0.001, ƞ^2^ = 0.41, power statistics = 0.95, and F_(1, 21)_ = 31.60, *p* < 0.001, ƞ^2^ = 0.60, power statistics = 0.99, and F_(1, 21)_ = 14.66, *p* = 0.001, ƞ^2^ = 0.41, power statistics = 0.95, respectively). For analyzed indicators, values were higher in the morning than in the evening in the experimental session, which might be interpreted as a decline in PS (*p* = 0.049, *p* = 0.013, and *p* = 0.037, respectively). Values of RangeML and RangeAP were also found to be higher in the morning in the experimental session (which might be interpreted as worse PS) than in the control session (*p* = 0.008 and *p* < 0.001, respectively).

The main effects of “session” were found for RangeAP (F_(1, 21)_ = 4.70, *p* = 0.042, ƞ^2^ = 0.18, power statistics = 0.54).

**Figure 4:**
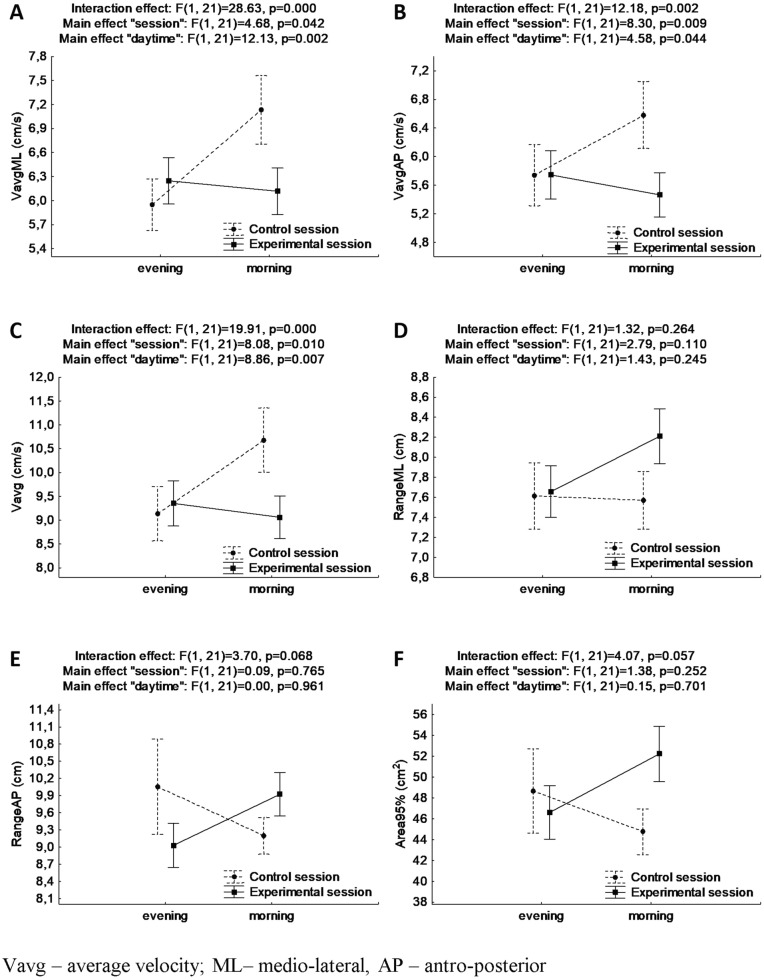
Mean and standard error of measurement values of postural stability indices in one-leg stance with eyes closed for evening and morning trials during control and experimental session, and results of two-way ANOVA with repeated measures (**A**, **B**, **C**, **D**, **E**, **F** for VavgML, VavgAP, Vavg, RangeML, RangeAP, Area95%, respectively).

Results for ANOVA with repeated measures for postural stability indicators in trials of OLS are presented in Fig. 4.

There were observed statistically significant interaction effects “session” × ”daytime” for VavgML, VavgAP and Vavg (F_(1, 21)_ = 28.63, *p* < 0.001, ƞ^2^ = 0.58, power statistics = 0.99, and F_(1, 21)_ = 12.18, *p* = 0.002, ƞ^2^ = 0.37, power statistics = 0.91, and F_(1, 21)_ = 19.91, *p* < 0.001, ƞ^2^ = 0.49, power statistics = 0.99, respectively). Bonferroni's detailed post-hoc analysis revealed lower values for each PS indicator in the morning in the experimental session (which might be interpreted as better PS) than in the control session (*p* < 0.001) as well as higher values in the morning than in the evening (which might be interpreted as worse PS) for the session without sleep deprivation (*p* < 0.001, *p* = 0.008 and *p* < 0.001, respectively).

For each PS indicator connected to COP velocity, significant main effects of “session” (F_(1, 21)_ = 4.68, *p* = 0.042, ƞ^2^ = 0.18, power statistics = 0.54, and F_(1, 21)_ = 8.30, *p* = 0.009, ƞ^2^ = 0.28, power statistics = 0.78, and F_(1, 21)_ = 12.13, *p* = 0.002, ƞ^2^ = 0.28, power statistics = 0.77, respectively) as well as main effects of “daytime” (F_(1, 21)_ = 28.63, *p* < 0.001, ƞ^2^ = 0.37, power statistics = 0.91, and F_(1, 21)_ = 4.58, *p* = 0.044, ƞ^2^ = 0.18, power statistics = 0.53, and F_(1, 21)_ = 8.86, *p* = 0.007, ƞ^2^ = 0.30, power statistics = 0.81, respectively) were found.

There were not any significant interaction effects “session” × ”daytime” as well as main effects for RangeML, RangeAP, and Area95%.

## Discussion

The study aimed to check the magnitude of SDep effects on PS among physically active young adults. Moreover, the issue of interest was to analyze the difference in PS change that occurs during the night—the evening-morning difference after sleep vs. the evening-morning difference after SDep.

First of all, it should be indicated that confusing results were obtained for evening measurements. Despite the fact they did not differ significantly, a clear tendency was observed for higher results in the control session, in the case of easier tasks (EO and EC). Potentially, this could be connected with the learning effect but previous studies demonstrated that this can occur only in more demanding tasks like during a narrow stance with eyes closed^49^ or standing on foam^50^ among young healthy subjects. Moreover, to reduce the possibility of this phenomenon, familiarization, randomization of trials order, and averaging results of two trials for each test version were applied in the current study. Another explanation is connected to the level of fatigue which is evidenced to affect PS^6^. Participants were asked not to change their activities. However, PA was not monitored in the study so it needs further investigation.

Taking into account the trends of change in both sessions, different reactions were noted, depending on the test versions. For EO, values of Vavg and VavgAP slightly increased in the morning in comparison to evening measurements in both sessions (significant main effect for “daytime” but no differences in post-hoc analysis). This finding is similar to other authors’ results. Halpern et al.^51^ reviewed this phenomenon and found no clear consensus for the daytime effect on postural control with slightly more favorable results obtained during the evening measurements^4^. However, some data indicate better results in the morning^52^, and in the afternoon^53^ as well as with no effect^54^. Halpern et al.^51^ concluded that existing discrepancies might be connected to the use of different posturographic platforms, the level of difficulty of posturographic tests, and whether or not the circadian rhythm is taken into account. For future research, they also recommended standardizing the type and timing of the test, with defined time frames. On the other hand, the observed slight increase in posturographic values cannot be directly interpreted as a worsening of PS, taking into account that postural sway is positive to some extent as proposed in the feedforward model^18,19^.

For EC, there were similar tendencies for average velocities in the experimental session. On the other hand, values of COP velocities in the morning in the control session significantly decreased in comparison to the evening measurements. In this study, subjects were not prevented from following their daily routines connected to classes or work. As they were physically active and had at least 1.5 h of intensive activity per day, one can expect higher values of COP displacements indicators in the evening—especially in more challenging tests as eyes closed^52^. It is possible, that for more challenging test versions (EC vs. EO) sleep plays a crucial role in regenerating the CNS and muscles after the fatigue effect increases during daily tasks. For example, Fullagar et al.^55^ noted that a reduction in the quality and quantity of sleep may result in an imbalance of the autonomic nervous system, simulating the symptoms of overtraining syndrome in athletes. In addition, sleep deprivation can also promote immune system dysfunction, hinder muscular recovery, and cause cognitive function decline through slower and less accurate cognitive performance. During sleep, muscles are deactivated by the brainstem^56^ to avoid activity while dreaming. Maybe this mechanism is also needed to achieve appropriate regeneration of muscles. However, the direct conclusion is limited because neither examination of the CNS nor physiological indicators of muscle function were used in this study.

In the case of ranges and surface area of COP displacements, higher values were found in the morning than in the evening in the experimental session both during EO and EC. Similar results were obtained by Ma et al.^3^. This might be interpreted as a slower or inadequate reaction to postural sway. The system “allows” for bigger deflections of COG from the optimum position and postural reactions are released with higher latency. However, it should be considered that among all time-domain COP parameters, only the sway path (directly related to average velocity in constant time of the measurement) is indicated as valuable in clinical practice, sufficiently reliable, and having discriminative power^57^.

Some explanations were proposed to explain what happens during a prolonged state of being awake. For instance, the role of attention is noted to affect PS^58^, and SDep is considered as a reason for the occurrence of lapses in attention^31^ according to the “lapse hypothesis” of Wilkinson^59^. Other authors also mentioned the possible role of factors connected to sight as reduced visual-spatial performance or sensitivity of visual perception^5^. Gomez et al.^31^ also concluded that during SDep, sensory integration might be slower or inappropriate, which impairs the ability to choose the most appropriate motor reaction to stabilize posture. Robillard et al.^12^ found that even if sensory or cognitive functions are unchallenged SDep had still an effect on PS. They speculated the possible importance of effectors or sensory inputs other than visual but this thesis needs further research. Another direction of explanation might be connected to the strength of lower limb muscles which is mentioned as a strong factor influencing PS^60^. It was observed that muscle strength was lowered during SDep^32,33^, which might be connected to lower body temperature after a night without sleep^34^.

During OLS an increase was observed in average velocities and a slight decrease in ranges and surface area of COP displacements between evening and morning measurements in the control session. This might be interpreted as lowering the time between the detection of COG displacement and the execution of postural reflexes in the morning. On the other hand, no changes in average velocities were noticed, but there was an increase in the spatial distribution of COP displacements in the experimental session. Similarly to the double stance, it might be connected to slower and inadequate postural reaction^31^. Additionally, it should be mentioned that a one-leg stance is quite a challenging test, especially among people during SDep. For instance, the area of COP displacements was 2–3 times greater than during double stance, and some of the participants were “close to falling” during tests. In this context, the obtained results might be explained by an increase in anxiety and increased stiffness of muscles^61,62^. The phenomenon is often observed in at-height workers and is interpreted as changing postural strategy to be more conscious in circumstances of danger^24,63^.

Taken together, it was found that changes in postural stability after SDep among subjects seemed to be rather typical when considering indirect comparison to previous research^4^. However, it should be remembered that slight differences in experimental protocols do exist between studies. During double stance, progressive changes may be observed depending on the difficulty of the task after SDep. The changes are first noted in the spatial distribution of COP displacements only, and then also in the velocity of displacements (EO and EC, respectively). On the other hand, the observed strong interaction effects are connected mainly to the difference in morning posturographic values between sessions, which generally indicate the opposite direction of changes between SDep and sleep. However, this should be carefully considered, given the mentioned shift between evening results. In the case of evening-morning comparison in the experimental session, there was a significant difference only in the case of ranges and surface area of COP displacements during EO and EC. Average velocities in all measurements as well as indicators of spatial distributions in OLS in the experimental session did not differ between evening and morning.

In conclusion, despite significant interaction effects, only results of spatial distribution indicators during double stance were higher in the morning than in the evening in the session after SDep. So, no clear decline of PS after SDep was observed. This may suggest that SDep prevents natural regeneration rather than significantly worsening postural stability among physically active adults. It is possible that systematic PA might be one of the factors decreasing the risk of accidents among people exposed to SDep.

## Limitations

It should also be mentioned that this work was limited. First, it was found differences in evening-morning changes connected to states of SDep and night with sleep, and the direction of these changes seems to be typical. However, as it was used a within-group study protocol^4,12^, it’s difficult to estimate if the magnitude of changes could differ according to higher PA levels. It is not well known whether inactive young adults with poor sleep quality experience more postural instability. Secondly, the participants were allowed to follow their daily routines during the day when the experiment started^5^. Next, PA was not monitored during the day when the experiment had started—however, a similar PA level was assumed since physical education students all follow such an exercise routine. Next, there were not any neurological and physiological examinations, so conclusions about disruptions in the CNS or increased muscle stiffness are indirect. Cognitive psychological tests like the Stroop test are lacking here which makes conclusions more speculative in the context of potential interaction between attention and PS in states of SDep. It is possible that a decrease in cognitive functions and less precise motor control due to lack of sleep could lead to a decrease in regional brain activity.

### Perspectives

Generally, further research is needed with a non-active control group. It would be also useful to monitor physical activity during the day as well as to estimate the level of fatigue or perceived exertion before and after the night of SDep. PS should be measured in additional time points on the day before and after examination to control daytime changes. It would be also reasonable to provide a study with measurements of PS in dynamic conditions. In further investigations, cognitive tests connected to attention should also be included. Moreover, previous research has shown that the effects of sleep deprivation on both postural control and sensory integration are age-dependent^12^. Expanding the studies to include the elderly seems to be justified. Furthermore, taking into account gender-based differences in neurological responses to SDep^64^ it would be reasonable to provide similar research in groups split according to gender.

## Data Availability

The datasets used and/or analysed during the current study available from the corresponding author on reasonable request.
